# Systemic ASBT inactivation protects against liver damage in obstructive cholestasis in mice

**DOI:** 10.1016/j.jhepr.2022.100573

**Published:** 2022-08-27

**Authors:** Roni F. Kunst, Dirk R. de Waart, Frank Wolters, Suzanne Duijst, Esther W. Vogels, Isabelle Bolt, Joanne Verheij, Ulrich Beuers, Ronald P.J. Oude Elferink, Stan F.J. van de Graaf

**Affiliations:** 1 Tytgat Institute for Liver and Intestinal Research, Amsterdam University Medical Centers, University of Amsterdam, Amsterdam, The Netherlands; 2 Amsterdam Gastroenterology, Endocrinology and Metabolism (AGEM), Amsterdam University Medical Centers, The Netherlands; 3 Department of Pathology, Amsterdam University Medical Centers, University of Amsterdam, Amsterdam, The Netherlands; 4 Department of Gastroenterology and Hepatology, Amsterdam University Medical Centers, University of Amsterdam, Amsterdam, The Netherlands

**Keywords:** Apical sodium-dependent bile acid transporter (ASBT), IBAT, NTCP, BSEP, PFIC, Alagille, Cholestasis, Bile salt pool size, Renal excretion, ALT, alanine transaminase, ASBT, apical sodium-dependent bile acid transporter, ASBTi, ASBT inhibitors, AST, aspartate transaminase, BDL, bile duct ligation, CCl_4_, carbon tetrachloride, CK7, cytokeratin 7, FRET, Förster resonance energy transfer, G-OCA, glycine-conjugated OCA, HepG2 cell, hepatocarcinoma cell, MDR2, multidrug resistance protein 2, NASH, non-alcoholic steatohepatitis, NGM282, non-tumorigenic fibroblast growth factor 19 analogue, NTCP, Na+/taurocholate cotransporting polypeptide, NucleoBAS, nuclear Bile Acid Sensor, OCA, obeticholic acid, PBC, primary biliary cholangitis, PentaOH, pentahydroxylated, RT-qPCR, real-time quantitative PCR, TCA, taurocholic acid, TetraOH, tetrahydroxylated, T-OCA, taurine-conjugated OCA, U2OS, osteosarcoma cell, UHPLC-MS, ultrahigh-performance liquid chromatography mass spectrometry, WT, wild-type

## Abstract

**Background & Aims:**

Non-absorbable inhibitors of the apical sodium-dependent bile acid transporter (ASBT; also called ileal bile acid transporter [IBAT]) are recently approved or in clinical development for multiple cholestatic liver disorders and lead to a reduction in pruritus and (markers for) liver injury. Unfortunately, non-absorbable ASBT inhibitors (ASBTi) can induce diarrhoea or may be ineffective if cholestasis is extensive and largely precludes intestinal excretion of bile acids. Systemically acting ASBTi that divert bile salts towards renal excretion may alleviate these issues.

**Methods:**

Bile duct ligation (BDL) was performed in ASBT-deficient (ASBT knockout [KO]) mice as a model for chronic systemic ASBT inhibition in obstructive cholestasis. Co-infusion of radiolabelled taurocholate and inulin was used to quantify renal bile salt excretion after BDL. In a second (wild-type) mouse model, a combination of obeticholic acid (OCA) and intestine-restricted ASBT inhibition was used to lower the bile salt pool size before BDL.

**Results:**

After BDL, ASBT KO mice had reduced plasma bilirubin and alkaline phosphatase compared with wild-type mice with BDL and showed a marked reduction in liver necrotic areas at histopathological analysis, suggesting decreased BDL-induced liver damage. Furthermore, ASBT KO mice had reduced bile salt pool size, lower plasma taurine-conjugated polyhydroxylated bile salt, and increased urinary bile salt excretion. Pretreatment with OCA + ASBTi in wild-type mice reduced the pool size and greatly improved liver injury markers and liver histology.

**Conclusions:**

A reduced bile salt pool at the onset of cholestasis effectively lowers cholestatic liver injury in mice. Systemic ASBT inhibition may be valuable as treatment for cholestatic liver disease by lowering the pool size and increasing renal bile salt output even under conditions of minimal faecal bile salt secretion.

**Lay summary:**

Novel treatment approaches against cholestatic liver disease (resulting in reduced or blocked flow of bile) involve non-absorbable inhibitors of the bile acid transport protein ASBT, but these are not always effective and/or can cause unwanted side effects. In this study, we demonstrate that systemic inhibition/inactivation of ASBT protects mice against developing severe cholestatic liver injury after bile duct ligation, by reducing bile salt pool size and increasing renal bile salt excretion.

## Highlights

• ASBT deficiency in mice markedly reduced bile duct ligation-induced liver injury.
• Systemic ASBT inactivation lowered total bile salt pool size.
• Systemic ASBT inactivation increased renal bile salt excretion.
• Reduced bile salt pool size before cholestasis onset improved liver injury.

## Graphical abstract

**Figure:**
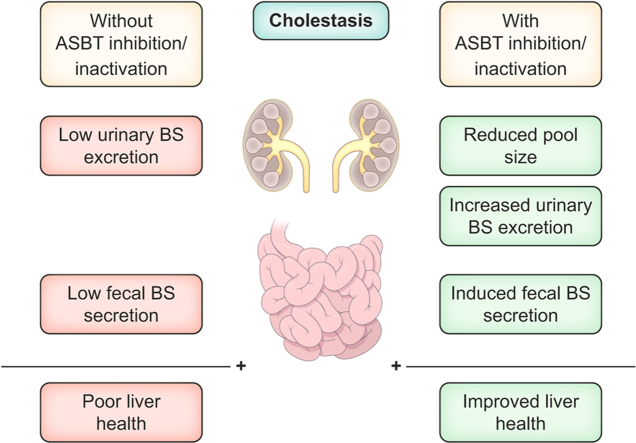

## Introduction

The apical sodium-dependent bile acid transporter (ASBT; also known as the ileal bile acid transporter [IBAT] or SLC10A2) is a key transporter in the enterohepatic circulation of bile salts and is expressed in various tissues such as the small intestine and kidneys, where it mediates bile salt uptake from the intestinal lumen and from primary urine, respectively. This prevents bile salts from being secreted in faeces or urine. Several studies have shown that interrupting the enterohepatic circulation can lower serum bile salt concentrations and reduce symptoms related to cholestasis, in both rodents and patients.[1], [2], [3], [4] Intestine-restricted ASBT inhibitors (ASBTi) effectively lower serum-conjugated bile acid concentrations and improve itching scores in patients with primary biliary cholangitis (PBC).^3^^,^^5^

Unfortunately, inhibition of ASBT leads to bile salt spilling over to the colon, which can result in adverse events such as diarrhoea, irregular bowel movement, and abdominal pain.^3^^,^[5], [6], [7] The fact that ASBT inhibition reduces intestinal farnesoid X receptor (FXR) activation, leading to lack of FGF19 signalling and derepressed/increased bile salt synthesis to compensate for the faecal bile salt loss, further contributes to the high colonic bile salt load. Conversely, in conditions where bile flow from the liver to the intestine is largely blocked, as in severe cholestasis, inhibition of intestinal bile salt uptake will not lower hepatic bile salt accumulation. Stimulation of renal bile salt excretion by inhibition of ASBT in the kidneys may be of benefit in such conditions. However, most ASBTi currently in clinical trials have minimal systemic exposure.

Here, we exploited the potential of systemic ASBT inhibition using a whole body ASBT knockout (KO) model. Remarkably, the consequence of ASBT deficiency has not been tested in experimental cholestatic conditions to date. One of the limitations of using ASBT KO mice as a model for systemic ASBT inhibition is that these mice have a smaller bile salt pool size and increased bile salt synthesis at the onset of cholestasis.^8^ Therefore, a second aim was to investigate the consequence of a lower bile salt pool size before the onset of cholestasis. To this end, we treated wild-type (WT) mice with an intestine-restricted ASBTi before inducing cholestasis. To further lower the bile salt pool size and simultaneously dampen the compensatory bile salt synthesis, we also used the Farnesoid X receptor (FXR) agonist obeticholic acid (OCA), which is expected to have largely left the enterohepatic circulation at the onset of the cholestasis with this experimental design. Finally, the role of ASBT in renal bile salt excretion during obstructive cholestasis was measured.

## Materials and methods

### Animals and experimental design

ASBT-deficient mice (ASBT KO) in a 129P2/OlaHsd background (Jackson, Bar Harbor, ME, USA) and littermate controls were bred and housed in the Academic Medical Center, Amsterdam, The Netherlands. Both male and female ASBT KO mice and WT littermates between 8 weeks and 4 months old were used to quantify total bile acid pool size (n = 7–8) or underwent complete common bile duct ligation (BDL) to study liver injury (n = 7–9). In this model, the gall bladder is ligated and removed. Furthermore, the common bile duct was ligated twice and cut in between to ensure that there is absolutely no bile flow from liver to the small intestine. Five days after BDL, mice were fasted for 6 h and anaesthetised using a mix of ketamine (100 mg/ml; Covetrus, Cuijk, The Netherlands) and xylazine (20 mg/ml; Covetrus, Cuijk, The Netherlands) in a dosage of 100 μl/10 g body weight before organs and tissues were collected. Similarly, ASBT-deficient mice and WT littermates in a FVB background (n = 3–4) were used for taurocholic acid (TCA; Sigma, Houten, The Netherlands) infusion with radiolabelled ^3^H-TCA (1 mCi/ml, PerkinElmer, Groningen, The Netherlands) and ^14^C-inulin (ARC 0124A-50, 0.1 mCi/ml, BioTrend, Cologne, Germany) via the jugular vein 2 days after BDL (n = 3–4). Infusion rate was increased every 30 min from 200 to 400 to 600 nmol/min∗100 g body weight TCA, and urine was collected directly from the urinary bladder using a cannula. ^3^H-TCA and ^14^C-inulin in urine were quantified in samples collected every 15 min using a liquid scintillation counter by adding Ultima Gold (PerkinElmer) directly to the urine samples. Two days after BDL, plasma (from the vena saphena) and urine (from the urinary bladder) samples were taken for ultrahigh-performance liquid chromatography mass spectrometry analysis (n = 4–5).

WT male mice (C57Bl/6JOlaHSD) were purchased from Envigo (Venray, the Netherlands) at 12 weeks old. Mice were fed a standard chow diet and treated for 2 days with a combination therapy of OCA (at 8 a.m.) and intestinal ASBTi (at 5 p.m.). Mice received 30 mg/kg body weight OCA (Selleckchem S7660, Bio-Connect B.V., Huissen, The Netherlands) or placebo in 1% methylcellulose and 10 mg/kg body weight intestine-restricted ASBTi (GSK2299027B^9^) or placebo in 0.5% hydroxypropyl methylcellulose and 0.1% Tween80 (AppliChem, Darmstadt, Germany) by oral gavage. On the third day, half of the mice were sacrificed to determine total bile salt pool size after combination therapy (n = 8 per group). The other half of the mice underwent BDL (n = 6–8 per group). The latter group was sacrificed 2 days after BDL without any further treatment to assess liver injury. All mice were cohoused with 2–5 mice per cage (individual housing occurred as an exception for faecal bile salt measurement) and kept on a 12-h light:12-h dark cycle with *ad libitum* access to food and water. All experiments have been performed at the Academic Medical Center. The study design and animal care and handling were approved by the Institutional Animal Care and Use Committee of the University of Amsterdam.

### Cell culture and treatment

All cells were cultured in normal DMEM (Lonza) supplemented with Bodinco 10% FBS (BioWest, Nuaillé, France), 1% pen/strep (Invitrogen, Landsmeer, The Netherlands), and 1% l-glutamine (Invitrogen, Landsmeer, The Netherlands). Serum was depleted from bile salts by charcoal before use. Cells were plated in a 96-well (cell viability) or 24-well plate (^3^H-TCA uptake and RT-qPCR) and grown into confluence before use.

### Cell viability assay

Human hepatocarcinoma cells (HepG2; ATCC, Manassas, VA, USA) were plated and grown into confluence, followed by a 24-h 1 μM OCA (Selleckchem S7660) and 1-h 4- or 2-mM carbon tetrachloride (CCl_4_, BDH 10074) treatment. Cell viability was measured by using Roche Cell Proliferation Reagent WST-1 (Sigma) according to the manufacturer’s protocol and spectrophotometrically quantified using the Synergy HTX Multi-Mode reader (BioTek/Agilent).

### Confocal microscopy-based FRET bile acid sensor

Human osteosarcoma cells (U2OS; ATCC, Manassas, VA, USA) stably expressing nuclear Bile Acid Sensor (NucleoBAS)^10^ were plated in 8-well coverslip-bottomed chamber slide (Thermo Fisher Scientific, 155411) and polyethylenimine (PEI; Brunschwig, Basel, Switzerland) transfected 24 h after plating with human Na+/taurocholate cotransporting polypeptide (NTCP or *SLC10A1*) cloned upstream of mKate2 (pmKate2-N1; Evrogen, Bio-Connect, Huissen, The Netherlands) or with human ASBT cloned in frame with a C-terminal SNAP tag (into pSNAPm; New England Biolabs, Ipswich, MA, USA). Forty-eight hours later, cells were imaged in Leibovitz L-15 medium (Gibco) with the 63× oil objective at a temperature-regulated (set at 37 °C) Leica SP8X-SMD confocal microscope (Leica microsystems) as described before.^11^ To test taurine-conjugated OCA (T-OCA) transport by NTCP, cells were incubated with 100 nM T-OCA (28243-1, Sanbio, Uden, The Netherlands), whereas 1.4 μM T-OCA was used for ASBT-dependent transport. Then, 5 μM GW4046 (Sigma) was used as a positive control to induce maximal FXR activation.

### ^3^H-TCA uptake assay

Human U2OS cells stably expressing human NTCP^12^ or human cholangiocarcinoma cell line CClP-1^13^ stably transduced with lentivirus encoding human ASBT (Vectorbuilder, Neu-Isenburg, Germany) were pre-incubated for 5 min with 10 μM OCA (Selleckchem S7660, Bio-Connect B.V., Huissen, The Netherlands), 10 μM glycine-conjugated OCA (G-OCA; Sanbio 28242-1), 10 μM T-OCA (Sanbio 28243-1), or any of the controls, 0.01% DMSO, 400 nM Myrcludex B (Pepscan, Lelystad, The Netherlands), or 4 μM GSK264W94 (GlaxoSmithKline). Next, 1 μM TCA containing ^3^H-TCA was added to the pre-incubation for 2 min, before intracellular ^3^H-TCA was measured using the liquid scintillation counter by adding cell lysate to Ultima Gold solution (PerkinElmer).

### RNA isolation and RT-qPCR

Tissues were snap-frozen in liquid nitrogen directly after isolation and stored at −80 °C until further processing. Tissue homogenisation was done using the Qiagen TissueLyser LT in stainless steel beads (Qiagen 69989) in TRI Reagent (Sigma). RNA integrity and concentration were measured using a NanoDrop ND-1000 UV/VIS Spectrophotometer (Thermo Scientific), and 1,000 ng was used for first-strand cDNA synthesis. RNA was treated with Roche DNAse (Sigma), followed by cDNA synthesis with Oligo-dT (Fermentas) and Revertaid reverse transcriptase (Fermentas). Real-time quantitative PCR (RT-qPCR) was performed on the BioRad CFX96 Touch Real-Time PCR with 5× diluted cDNA and Bioline SensiFAST SYBR No ROX kit (GC Biotech, Waddinxveen, The Netherlands). Results were processed using the BioRad CFX Maestro 5.0 software and LinRegPCR 12.5 software and excluded when they failed quality checks.^14^ In addition, if a plateau was not reached at cycle 39, gene expression was not detected (ND), and value 0 was used for statistics. Primers used for RT-qPCR are shown in Table S1.

### Bile salt analysis

Bile salt concentrations in urine, blood plasma, faeces, the small intestine, and the liver were measured by reverse-phase HPLC as described before.^15^ Total bile salt pool size is calculated as the sum of bile salts measured in the small intestine, liver, and gall bladder. Sulfated and polyhydroxylated bile salts were measured by ultrahigh-performance liquid chromatography mass spectrometry (UHPLC-MS) based on a method described earlier.^16^ Samples were analysed in a Thermo Scientific Dionex UltiMate 3000 UHPLC coupled to a Bruker ultrahigh-resolution trapped ion mobility spectrometry time-of-flight (UPLC-timsTOF) mass spectrometer. Chromatographic separation occurred by a HSS T3 column (Waters, 1.8 μm particle size, 2.1 mm × 150 mm) and a mobile phase containing (A) water with 0.1% formic acid and 10 mmol/L ammonia and (B) methanol:isopropanol 1:1 (v/v). The gradient was 0% B and 100% A at 0 min, ramped up to 20% B at 1 min, 70% B at 15 min, and 100% B at 18 min. It was then held for 2 min at 100% B before it went back to 0% B and 100% A at 20.1 till 21 min. Flow was set to 0.4 ml/min. Whole plasma and urine samples were spiked with 5 μM internal standard 5(S)-HETE-D_8_ (Sanbio) and processed for analysis. Samples were precipitated by adding 5 volumes of ice-cold acetonitrile while being vortexed, and supernatant was transferred to the SpeedVac vacuum concentrator (Eppendorf) until dry. In addition, urine samples were freeze-dried with the help of liquid nitrogen. Dried samples were reconstituted in 25% methanol, sonicated at 4 °C, and centrifuged at maximum speed, 4 °C, before the supernatant was measured. Final results were corrected for volume and internal standard after measurement. Data are shown as the sum of peak ion intensities of indicated retention times at expected *m*/*z* 530.28 ± 0.02, 546.27 ± 0.02, and 594.24 ± 0.02 for taurine-conjugated tetrahydroxylated, pentahydroxylated, and sulfated bile salts, respectively. No concentrations were calculated owing to the absence of standards of the aforementioned bile salts.

### Histology and immunohistochemistry

Mouse liver tissues were directly fixed in 3.7% paraformaldehyde (Merck) for 24 h and stored in 70% ethanol before paraffin embedding and 4.5 μm sectioning using the Rotary Microtome Microm HM 340E (Thermo Scientific). Hematoxilin (MilliporeSigma, 51275) and eosin (MilliporeSigma, E4382) (H&E) staining was then performed. Anti-cytokeratin 7 (CK7) staining was performed on liver sections blocked with normal antibody diluent (ImmunoLogic) and incubated with (1:8,000) rabbit anti-CK7 (Abcam, ab181598) overnight at 4°C. After incubation with BrightVision anti-IgG poly-horse radish peroxidase-conjugated secondary antibody (ImmunoLogic), Vector NovaRED peroxidase substrate (Brunschwig, Basel, Switzerland) was used to visualise horse radish peroxidase. Digital imaging of the section was done using an Olympus BX-51 microscope (Olympus), equipped with a 20× objective. Tile scans were made using the 5× objective of the Leica DM600B microscope, and necrotic areas were scored as percentage (surface) of the total liver section using ImageJ. CK7 positive stain was quantified by colour deconvolution using ImageJ FIJI.

### Plasma biochemistry

Plasma biomarkers for liver injury and cholestasis, alanine transaminase (ALT), aspartate transaminase (AST), alkaline phosphatase, and bilirubin were 5 or 10 times diluted in 0.9% NaCl and measured by routine clinical biochemistry testing at the LAKC, Amsterdam Medical Center. These biomarkers are measured using photometric assay tests on the Roche Cobas c502/702 analyser (Roche Diagnostics).

### Statistics

Data are provided as mean ± SD. Individual data points are shown and represent different mice or cages in case of the faecal bile salt data. Differences between 2 groups are calculated by an unpaired parametric Student’s *t* test. Comparisons between >2 groups are made using a 1-way ANOVA, followed by Tukey’s (WST-1 cell viability) or Dunnett’s (^3^H-TCA uptake *in vitro*) multiple comparisons. A *p* value ≤0.05 is considered to be statistically significant and is calculated using GraphPad Prism v9.0 (GraphPad Software Inc, La Jolla, CA, USA). Group size was determined per experiment with a common SD of 15%, a power of 80%, and a *p* value cut-off of 0.05.

Further details about animals, cell lines, chemicals, and plasmids used can be found in the Supplementary CTAT Table.

## Results

### Deficiency of ASBT has hepatoprotective effects after BDL in mice

**Fig. 1:**
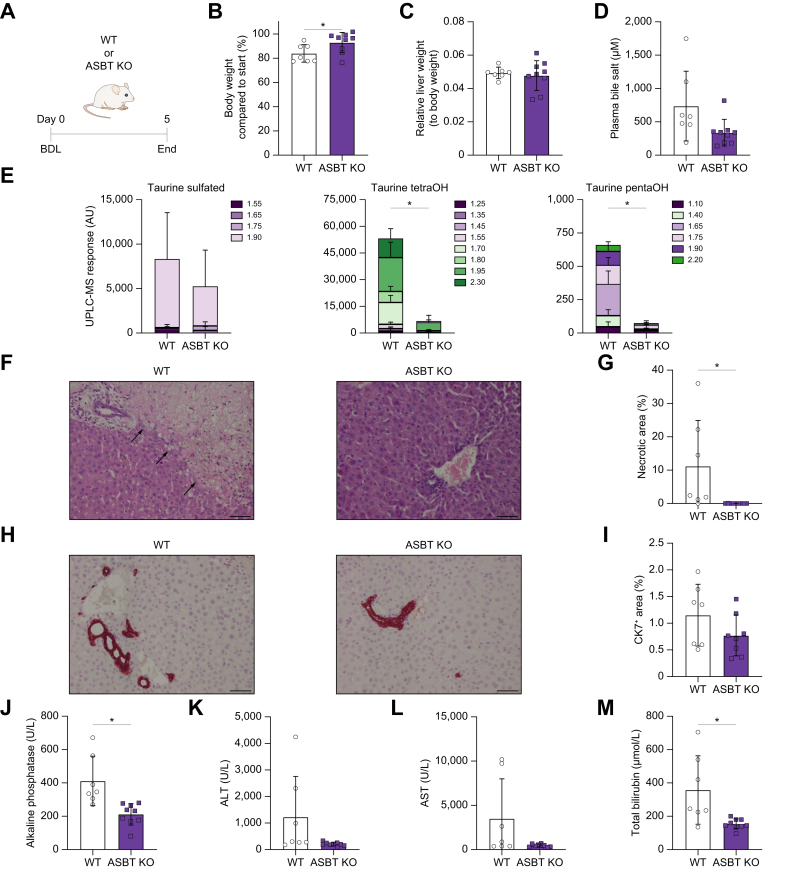
Systemic ASBT deficiency protects mice against cholestatis-induced liver injury. (A) Schematic experimental overview. (B) Body weight change after BDL. (C) Liver-to-body weight ratio after BDL. (D) Plasma bile salt concentration after BDL. (E) Plasma taurine-conjugated sulfated, taurine-conjugated tetrahydroxylated, and taurine-conjugated pentahydroxylated bile salts measured by UPLC-MS. Data are shown as the sum of peaks at indicated retention times for the same mass ± SD. n = 4–5 mice. (F) 200× liver H&E stain with arrows indicating necrotic areas; the scale bar represents 50 μm. (G) Liver necrotic area quantification. (H) 200× liver CK7 stain; the scale bar represents 50 μm. (I) Liver CK7 quantification. (J) Plasma alkaline phosphatase. (K) Plasma ALT. (L) Plasma AST. (M) Plasma bilirubin. Significance was determined using a unpaired parametric Student’s *t* test. Individual values are shown ± SD. ∗*p* <0.05. ALT, alanine transaminase; ASBT, apical sodium-dependent bile acid transporter; AST, aspartate transaminase; BDL, bile duct ligation; CK7, cytokeratin 7; KO, knockout; pentaOH, pentahydroxylated; tetraOH, tetrahydroxylated; WT, wild-type.

Severe extrahepatic cholestasis was induced in adult WT or ASBT KO mice by performing BDL to interrupt any bile flow to the small intestine (Fig. 1A). As a result, bile salts can only leave the systemic circulation via the urine. Body weight loss of ASBT KO mice after BDL was attenuated compared with WT mice (Fig. 1B). After 5 days, WT mice reached humane endpoint (∼20% body weight loss), whereas ASBT KO mice only lost ∼10% body weight compared with that pre-BDL. Liver-to-body weight ratio was not different in mice with ASBT deficiency after BDL (Fig. 1C). Plasma bile salt concentrations were numerically decreased in ASBT KO mice after BDL but not statistically different from WT (Fig. 1D). Furthermore, UPLC-MS analysis showed varying results in taurine-conjugated sulfated bile salt presence in both WT and ASBT KO mice 2 days after BDL but lowered taurine-conjugated tetrahydroxylated and pentahydroxylated bile salts in ASBT KO mice (Fig. 1E). Compared with livers of ASBT KO mice, those of WT mice displayed more extensive (confluent) necrosis of hepatocytes (*p* = 0.03) 5 days after BDL but also displayed some portal oedema and minor bile duct damage (Fig. 1F and G). By contrast, ASBT KO livers, in general, did not have any signs of necrosis or fibrosis but did have minor bile duct damage. CK7, a sensitive marker for bile duct injury, was not different between WT and ASBT KO mice as this model primarily induced confluent lobular and parenchymal damage (Fig. 1H and I). Reduced gene expression of inflammatory marker *Mcp-1* but no change in fibrotic and/or inflammatory markers *α-Sma*, *Col1a1*, and *Tnfα* (Fig. S1A–D) was found in livers of ASBT KO mice compared with those of WT mice after BDL. Expression of FXR-dependent small heterodimer particle (*Shp*) was not changed between ASBT KO and WT mice, whereas *Cyp7a1*, the rate-limiting enzyme in bile salt synthesis, was increased in bile duct-ligated ASBT KO mice (Fig. S1E and F). Bile salt transporters expressed in the liver were not changed, except for lower expression of *Slc51b* (encoding OSTβ) in ASBT KO mice (Fig. S1G–I). Five days after BDL, alkaline phosphatase levels were decreased by 49% in ASBT KO mice, demonstrating that systemic absence of ASBT has hepatoprotective effects (Fig. 1J). Plasma ALT (*p* = 0.06) and AST (*p* = 0.06) followed this trend, and plasma bilirubin levels decreased by 57% (Fig. 1K–M).

### ASBT deficiency reduces total bile salt pool size and increases urinary bile salt excretion

**Fig. 2:**
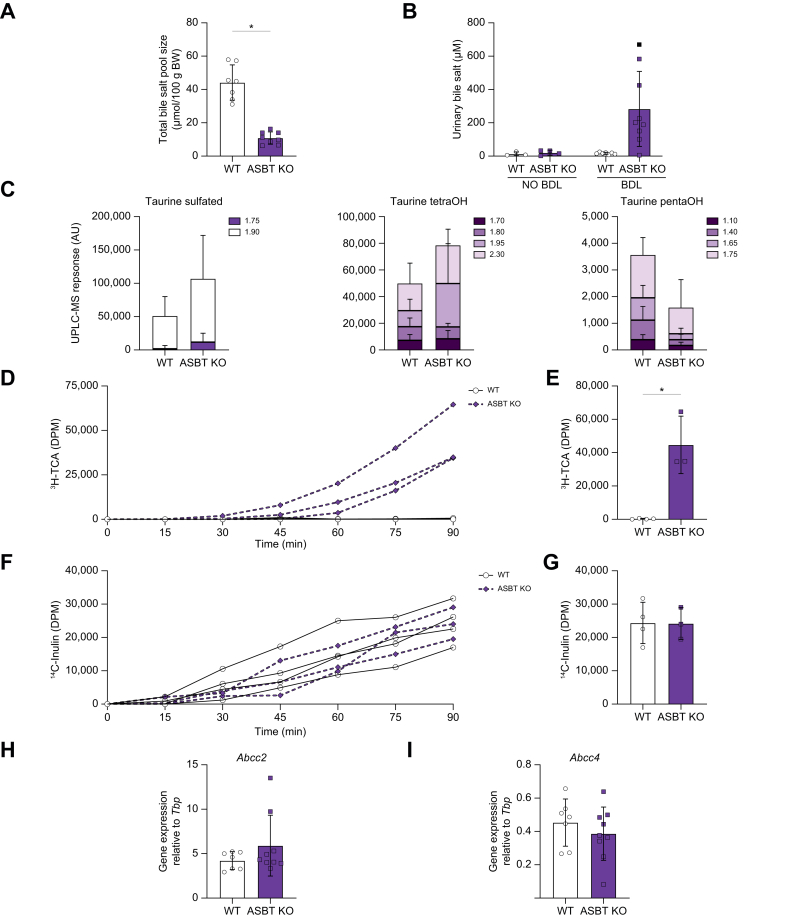
ASBT-deficient mice have reduced total bile salt pool size and increased renal bile salt excretion. (A) Total bile salt pool size. (B) Urinary bile salt excretion. (C) Urine taurine-conjugated sulfated, taurine-conjugated tetrahydroxylated, and taurine-conjugated pentahydroxylated bile salt measured by UPLC-MS. Data are shown as the sum of peaks at indicated retention times for the same mass ± SD. n = 4–5 mice. (D) Urinary ^3^H-TCA excretion in a 200–600 nmol/min∗100 g body weight TCA infusion experiment. (E) ^3^H-TCA in urine at t = 90 min. (F) Complementary urinary ^14^C-inulin excretion. (G) ^14^C-inulin in urine at t = 90 min. (H) Kidney *Abcc2* mRNA expression. (I) Kidney *Abcc4* mRNA expression. Significance was determined using a unpaired parametric Student’s *t* test. Individual values are shown ± SD. ∗*p* <0.05. ASBT, apical sodium-dependent bile acid transporter; BDL, bile duct ligation; KO, knockout; pentaOH, pentahydroxylated; TCA, taurocholic acid; tetraOH, tetrahydroxylated; WT, wild-type.

To explain the differences in the liver health of the WT and ASBT KO mice after inducing severe obstructive cholestasis, we first quantified the total bile salt pool size. In line with previous observations from Dawson *et al.*,^8^ we found that ASBT KO mice have a decreased bile salt pool size in control (no BDL) conditions (Fig. 2A). Furthermore, WT mice had a more hydrophilic bile salt pool than the ASBT KO. Because bile salts leave the enterohepatic circulation via either the faeces or urine, we quantified urinary bile salt excretion in WT and ASBT KO mice, with or without BDL. In healthy conditions (no BDL), urinary bile salt excretion was virtually absent in both WT and ASBT KO mice (Fig. 2B). After BDL, most of the ASBT KO mice displayed increased bile salt concentrations in urine, whereas bile salt levels in urine of WT mice was similar with or without BDL. Taurine-conjugated sulfated or tetrahydroxylated/pentahydroxylated bile salts in urine after BDL were not statistically different in ASBT-deficient mice compared with those in WT mice (Fig. 2C). To provide a more quantitative assessment of renal bile salt excretion, additional experiments were performed in bile duct-ligated ASBT KO and WT mice. After 90-min infusion of TCA, spiked with ^3^H-TCA and ^14^C-Inulin, against an increasing gradient, WT mice did not induce bile salt excretion via the urine, whereas ASBT KO mice did (Fig. 2D and E). ^14^C-Inulin excretion was similar in both groups, suggesting that the glomerular filtration rate, and thus kidney function, of ASBT KO and WT mice is comparable (Fig. 2F and G). In addition, renal expression of bile salt transporters MRP2 (gene *Abcc2*) and MRP4 (gene *Abcc4*) was not affected by ASBT deficiency, suggesting that the increase in urinary bile salt excretion is a direct consequence of ASBT deficiency (Fig. 2H and I).

### Reduced pool size before onset of cholestasis alleviates cholestasis-induced liver injury in mice

**Fig. 3:**
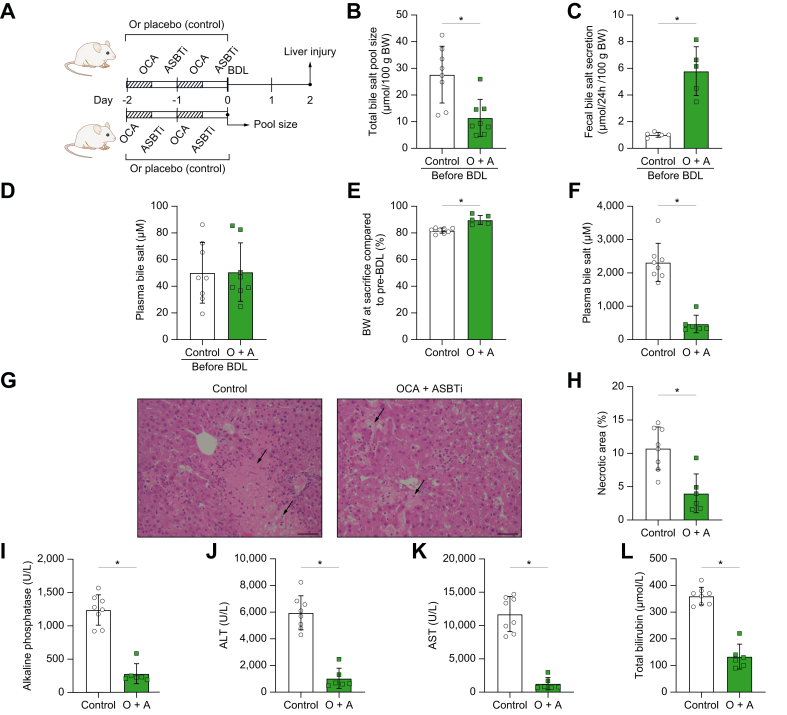
Reduced bile salt pool size before the onset of cholestasis has hepatoprotective effects. (A) Schematic experimental overview; mice received 2-day oral gavage with OCA + ASBTi or control before they underwent BDL or were sacrificed to determine pool size (B) Total bile salt pool size before BDL (C) Faecal bile salt secretion before BDL. (D) Plasma bile salt concentration before BDL. (E) Body weight change after BDL. (F) Plasma bile salt concentration after BDL. (G) 200× liver H&E stain with arrows indicating necrotic areas; the scale bar represents 50 μm. (H) Liver necrotic area quantification. (I) Plasma alkaline phosphatase. (J) Plasma ALT. (K) Plasma AST. (L) Plasma bilirubin. Significance was determined using a unpaired parametric Student’s *t* test. Individual values are shown ± SD. ∗*p* <0.05. ALT, alanine transaminase; ASBTi, apical sodium-dependent bile acid transporter inhibitors; AST, aspartate transaminase; BDL, bile duct ligation; BW, body weight; OCA, obeticholic acid.

As we and others have shown that ASBT deficiency leads to a reduced bile salt pool size, we next wanted to determine if this could cause the hepatoprotective effects seen during cholestasis. To study this, we started a 2-day treatment of OCA to repress bile salt synthesis and intestine-restricted ASBTi to increase faecal bile salt secretion (Fig. 3A). This, we hypothesised, would lead to a strong reduction in bile salt pool size while limiting enterohepatic circulation of OCA. After 2 days of treatment, the total bile salt pool size of the treatment group was reduced by ∼59% (*p* = 0.003) compared with that of the placebo-treated controls, consistent with our hypothesis (Fig. 3B). The combination treatment of OCA + ASBTi increased faecal bile salt secretion (Fig. 3C); however, plasma bile salt levels were not affected by treatment (Fig. 3D). Liver gene expression of *Cyp7a1* was increased, whereas ileal *Fgf15* expression tended to be decreased, most likely a reflection of the final ASBTi dose that was given the evening before sacrifice (Fig. S2A and B). In addition, expression of *Fabp6* (encoding IBABP) or any of the bile salt transporters *Slc10a2* (encoding ASBT), *Slc51a* (encoding OSTα), and *Slc51b* did not change as a result of treatment in line with modest/absent systemic OCA exposure during the second part of the experiment, as described below (Fig. S2C–F).

Having confirmed that the 2-day treatment with OCA and ASBTi effectively reduced the bile salt pool size, BDL was performed to induce cholestasis. From the day of BDL until the end of the experiment, mice did not receive any treatment to specifically study the effect of a reduced bile salt pool size at the onset of cholestasis. Two days after BDL, organs and tissues were collected to assess liver injury. Mice with a reduced bile salt pool size caused by OCA + ASBTi pretreatment were protected against severe weight loss after BDL (Fig. 3E). Two days after BDL, both groups showed minimal bile duct damage, (peri)portal oedema, and numerous patchy necrotic areas of liver parenchyma (Fig. 3F). However, mice with OCA + ASBTi pretreatment had less liver necrotic areas (*p* = 0.002; Fig. 3G). In line with this, plasma alkaline phosphatase, AST, and ALT were dramatically decreased compared with placebo controls, suggesting improved liver health (Fig. 3I–K). Plasma bilirubin was also lower in the OCA + ASBTi treatment group (Fig. 3L). Inflammatory and/or fibrotic markers in the liver were not affected by OCA + ASBTi pretreatment (Fig. S3A–D). There were no differences in expression levels of *αSma, Mcp-1, Col1a1*, and *Tnfα*. Mice with reduced pool size before BDL had increased liver *Cyp7a1* expression compared with control mice after BDL, confirming the minimal systemic OCA presence during the BDL phase of the experiment. Liver *Shp* and *Slc51b* remained unaltered, whereas *Abcb11* (encoding BSEP) and *Slc10a1* were more abundant in the treatment group (Fig. S3F–I). In the Ileum, gene expression of *Fgf15, Slc10a2, Fabp6, Slc51a*, and *Slc51b* was decreased in the OCA + ASBTi pretreatment group (Fig. S3J–N).

### OCA conjugates are substrates for the bile salt transporters ASBT and NTCP

**Fig. 4:**
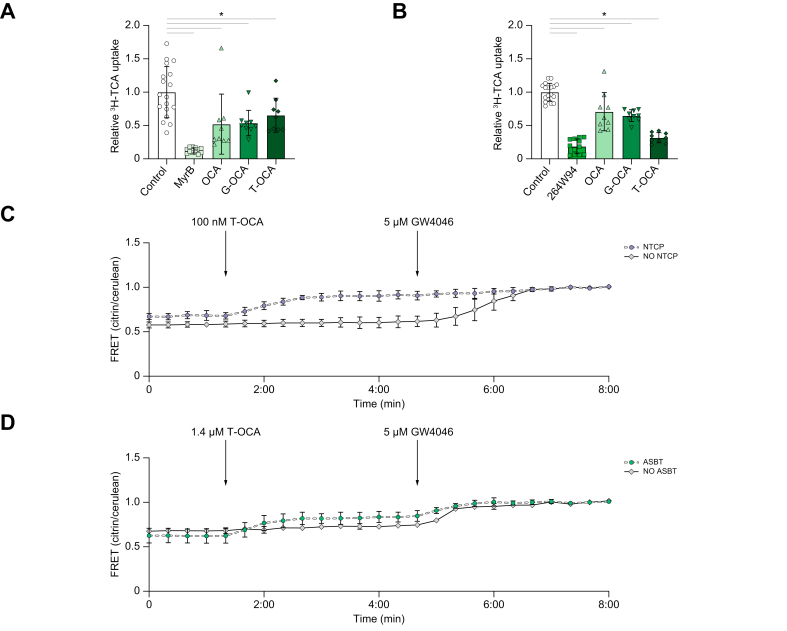
T-OCA is a substrate for bile salt transporters NTCP and ASBT. (A) Relative ^3^H-TCA uptake via NTCP in the presence of 0.01% DMSO, 400 nM Myrcludex B, 10 μM OCA, 10 μM G-OCA, or 10 μM T-OCA. (B) Relative ^3^H-TCA uptake via ASBT in the presence of 0.01% DMSO, 4 μM GSK264W94 B, 10 μM OCA, 10 μM G-OCA, or 10 μM T-OCA. (C) FRET signal (citrin/cerulean ratio) after addition of 100 nM T-OCA and 5 μM GW4046 to U2OS_NucleoBAS cells with and without NTCP. (D) FRET signal (citrin/cerulean ratio) after addition of 1.4 μM T-OCA and 5 μM GW4046 to U2OS_NucleoBAS cells with and without ASBT. Results of the FRET assay are shown as mean ± SD (n = 4–10 individual cells). Relative ^3^H-TCA uptake data are presented as mean ± SD of n = 3 experiments with 3–6 wells per condition, corrected for protein content. Significance was determined using a 1-way ANOVA followed by Dunnett’s multiple-comparisons analysis. ∗*p* <0.05. ASBT, apical sodium-dependent bile acid transporter; FRET, Förster resonance energy transfer; G-OCA, glycine-conjugated OCA; MyrB, Myrcludex B; NucleoBAS, nuclear Bile Acid Sensor; OCA, obeticholic acid; T-OCA, taurine-conjugated OCA; U2OS, osteosarcoma cell.

In our current experimental set-up *in vivo*, we alternated dosing of OCA and an ASBTi. It is unclear how OCA enters the cells and whether OCA-induced FXR agonism is bile salt transporter dependent. TCA uptake by NTCP or ASBT is reduced respectively by 48 and 30% in the presence of OCA (Fig. 4A and B). Incubation of NTCP- or ASBT-expressing U2OS cells with G-OCA or T-OCA showed similar reductions in TCA uptake. In particular, T-OCA is a strong competitor for bile salt uptake via ASBT, as TCA uptake was lowered to 32% in the presence of T-OCA. To actually demonstrate (conjugated) OCA transport, *in vitro* experiments were performed using a Förster resonance energy transfer (FRET) sensor for bile acid based on the FXR ligand binding domain.^10^ Incubation of U2OS cells expressing either NTCP or ASBT together with this bile acid sensor showed an increased FRET ratio upon T-OCA exposure in the nM or μM range, respectively (Fig. 4C and D). Cells that did not express any of the bile salt transporters did not display an increased FRET ratio, suggesting that T-OCA requires bile salt transporters to enter the cell. Addition of GW4064, a cell-permeable synthetic FXR agonist, at the end rapidly induced FRET changes in the cells without NTCP or ASBT expression, whereas the sensor was already (virtually) maximally activated by T-OCA in ASBT/NTCP-expressing cells. Of note, unconjugated OCA did result in increased activation of the FXR-based FRET sensor for bile salt in the absence of a bile salt transporter, likely entering via diffusion into the cells (not shown).

### Hepatoprotective effects as a result of OCA + ASBTi treatment are a direct consequence of reduced toxic bile acid accumulation

**Fig. 5:**
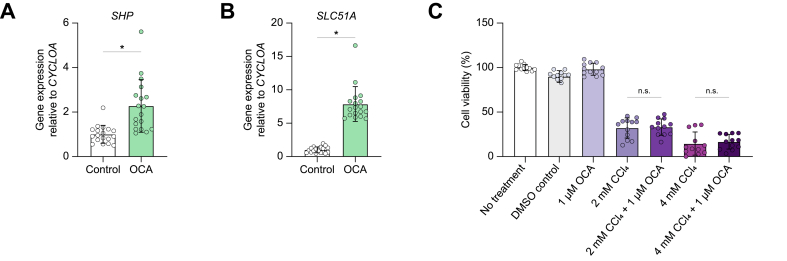
OCA treatment upregulates FXR target genes but does not protect against CCl_4_-induced toxicity in HepG2 cells. (A) mRNA expression of *SHP.* (B) mRNA expression of *SLC51A*. (C) WST-1 cell viability assay after 1-h incubation with CCl_4_. For RT-qPCR, mean ± SD of n = 3 experiments with sextuplets is shown. For WST-1 cell viability, mean ± SD of n = 3 experiments with quadruplicates is shown. Significance was determined using a 1-way ANOVA followed by Tukey’s multiple-comparisons analysis. ∗*p* <0.05. CCl_4_, carbon tetrachloride; FXR, farnesoid X receptor; HepG2 cell, hepatocarcinoma cell; OCA, obeticholic acid; RT-qPCR, real-time quantitative PCR; *SHP*, small heterodimer particle.

Lastly, to test whether OCA treatment is directly responsible for the improved liver health of the mice treated with OCA + ASBTi or indirectly by changing the bile salt pool size, *in vitro* experiments were performed in a CCl_4_-induced toxicity model. This model is independent of bile salt/cholestasis-induced toxicity. HepG2 cells showed increased expression of FXR target genes *SHP* and *SLC51A* after 24-h OCA treatment (Fig. 5A and B). Subsequently, a toxic environment was created by the addition of CCl_4_. Cell viability rapidly decreased but was independent of OCA pretreatment (Fig. 5C). Taken together, this suggests that the hepatoprotective effects seen *in vivo* are cholestasis dependent.

## Discussion

This study demonstrates that mice with systemic deficiency of ASBT activity displayed reduced liver damage in a model for obstructive cholestasis. In this model, faecal bile salt secretion is virtually absent, and the hepatoprotective effects observed may be assigned to the increased bile salt excretion in urine. Increased urinary bile salt excretion has already been shown to have hepatoprotective effects,^17^ a finding that is strengthened by our study. By contrast, the enhanced bile salt loss in ASBT-deficient mice also leads to a reduced total bile salt pool size.^8^ Reducing the bile salt pool size and increasing the renal bile salt excretion improved liver health in cholestatic *Slc51a*-deficient mice.^18^ Renal *Slc10a2* expression was downregulated, whereas ASBT protein was not detected in *Slc51a*-deficient mice after BDL, proving the ability of the system to adapt and prevent severe cholestasis-induced injury. Furthermore, a study in rats showed a downregulation of ASBT function already 24 h after BDL, whereas protein levels remained similar.^19^ This is a long-lasting effect as, 24 days after BDL, *Slc10a2* was still downregulated compared with no-BDL controls.^20^

Multiple strategies to reduce the bile salt load in the liver have been tested in both preclinical and clinical models. Increased faecal bile salt secretion by intestinal ASBT inhibition improves itching scores in patients with PBC.^3^^,^^5^ *In vivo*, 4-week intestine-restricted ASBT inhibition (meaning only inhibition in the intestine) improved serum alkaline phosphatase and ALT levels and lowered serum bile salt concentrations in the *Mdr2-*deficient mouse.^1^ In the same model, repressing bile salt synthesis via non-tumourigenic fibroblast growth factor 19 (FGF19) analogue NGM282 improved liver histology, reduced inflammation, and lowered serum alkaline phosphatase, ALT, and AST concentrations.^21^ *Mdr2-*deficient mice have a reduced hepatic bile salt pool and reduced serum bile acid levels after treatment with NGM282. In this study, we reduced the total bile salt pool by combining 2 promising strategies of reducing bile salt synthesis (OCA) and stimulating faecal bile salt secretion (intestine-restricted ASBTi).^22^ More recently, it was shown that 6 weeks of OCA treatment dampens inflammasome activation in a non-alcoholic steatohepatitis (NASH) mouse model and might thus contribute to improved liver health independent of cholestasis.^23^ In contrast, OCA treatment *in vitro* in a CCl_4_ toxicity model did not show any differences in cell viability, suggesting that the short-term hepatoprotective effects of the 2-day pretreatment with OCA + ASBTi are cholestasis/bile salt dependent. We also demonstrate that T-OCA (similar results for G-OCA; data not shown) is a substrate for both ASBT and NTCP. We used this finding and combined OCA treatment with ASBTi to lower the bile salt pool size while ensuring minimal OCA exposure after stopping the treatment. In our model, we found elevated liver *Cyp7a1* after 2-day treatment with OCA + ASBTi as a result of the ASBTi treatment, suggesting that FXR activation by OCA is indeed not sustained. This also implies that for effective OCA + ASBTi combination treatment in cholestatic conditions, as we proposed earlier specifically for primary sclerosing cholangitis,^22^ adequate timing of dosing both compounds is extremely important to reaching sufficient efficacy of both treatments.

Recently, it was shown that *Mdr2-*deficient mice that also lack *Bsep* have increased bile salt hydroxylation, and thus a more hydrophilic and less toxic bile salt pool, and also the total bile salt pool was reduced compared with that of the MDR2 KO.^24^^,^^25^ Together, this resulted in protection against severe liver injury. Increased levels of bile salt polyhydroxylation does not seem to contribute to beneficial consequences of ASBT inactivation as ASBT KO mice have reduced plasma taurine-conjugated tetrahydroxylated and pentahydroxylated bile salts, and there is no increase in excretion of these bile salts in urine.

Both liver histology and gene expression showed increased severity of disease in WT mice compared with ASBT KO mice as a result of obstructive cholestasis. The increase in urinary bile salt excretion likely plays a role in the hepatoprotective effects observed in this animal model. This is particularly relevant in severe cases of cholestasis, where intestine-restricted ASBTi may be a less effective strategy. It has been reported that renal bile salt transporters *Abcc2* and *Abcc4* are upregulated in cholestatic conditions^18^; however, no difference in gene expression was found between WT and ASBT KO mice after BDL (Fig. 2H).

Ongoing clinical studies are primarily focused on intestinal ASBT inhibition, as this forms by far the largest contributor in bile salt excretion from the body in normal conditions. However, as shown here in *in vivo* infusion experiments, renal ASBT inhibition can increase urinary bile salt concentration ∼17-fold and excretion in infusion studies up to ∼170-fold, thereby proving the rationale of developing and testing systemic ASBT inhibitors. Increased renal bile salt excretion induced by systemic ASBTi might further contribute to lowering colonic bile salt load and ameliorate the associated unwanted side effects.

Taken all together, this study suggests that novel ASBT-targeted therapeutic strategies against liver cholestatic diseases should include not only intestine-restricted compounds but also systemically acting compounds. These may lead to enhanced renal bile salt excretion as an extra factor resulting in a lower bile salt pool size. Finally, and in line with previously described strategies/conditions that lead to a reduced bile salt pool size at the onset of experimental cholestasis, a combination of transient FXR agonism and ASBT inhibition dampens cholestasis-induced liver damage.

## Financial support

This work was supported by grants from The Netherlands Organization for Scientific Research (Vidi 91713319 and Vici 09150182010007 to SFJVDG) and the 10.13039/100010663European Research Council (Starting grant 337479 to SFJVDG).

## Authors’ contributions

Carried out experiments: RFK, DRDW, EWV. Performed mouse surgeries: SD, IB. Analysed liver histology: JV. Performed UPLC-MS analysis: FW. Developed the study concept and design: RFK, RPJOE, UB, SFJVDG. Drafting and initial review of the manuscript: RFK, DRDW, FW, SD, EWV, IB, JV, UB, RPJOE, SFJVDG. Obtained funding: SFJVDG. Were involved in the analysis and interpretation of data and have read and approved the manuscript: All authors.

## Data availability statement

All data files are available upon request.

## Conflicts of interest

Ambys consultancy to institution of SFJVDG. The other authors report no conflicts of interest.

Please refer to the accompanying ICMJE disclosure forms for further details.
