# Supplementary material for: Systemic ASBT inactivation protects against liver damage in obstructive cholestasis in mice
Source: JHEP Rep. 2022 Aug 27;4(11):100573. doi: 10.1016/j.jhepr.2022.100573 (PMC9494276; doi:10.1016/j.jhepr.2022.100573)
Supplement: Multimedia component 1 [file mmc1.pdf]

Systemic ASBT inactivation protects against liver damage in obstructive cholestasis in mice

Roni F. Kunst, Dirk R. de Waart, Frank Wolters, Suzanne Duijst, Esther W. Vogels, Isabelle Bolt, Joanne Verheij, Ulrich Beuers, Ronald P.J. Oude Elferink, Stan F.J. van de Graaf

Table of contents

Table S1..2

Fig. S1..3

Fig. S2..4

Fig. S3..5

Supplementary references...6

Table S1. RT-qPCR primers for gene expression.

Gene	FW (5→ 3)	RV (5→ 3)	Amplicon
m_Hprt	TTGCTCGAGATGTCATGAAGGA	AGCAGGTCAGCAAAGAACTTATAG	91
m_Tbp	GGAGAATCATGGACCAGAACA	GATGGGAATTCCAGGAGTCA	89
m_CycloB	TCGGAGCGCAATATGAAGGT	AAAAGGAAGACGACGGAGCC	65
m_Slc10a1	TGGCTACCTCCTCCCTGATG	GCCAGGTTGTGTAGGAGGAT	380
m_Slc10a2	GGGGTATCTTCGTGGGCTTC	TGCTAACACTGAGGTCCATGTC	189
m_Abcb11	TGGAAAGGAATGGTGATGGG	CAGAAGGCCAGTGCATAACAGA	77
m_Slc51a	GGCATCTATGACCCAGGAGA	TGGATCCCATGTTCTGTTCA	151
m_Slc51b	GACCACAGTGCAGAGAAAGC	ATTCCAAGGAGCCGCATCT	102
m_Tnfa	CATCTTCTCAAAATTCGAGTGACAA	TGGGAGTAGACAAGGTACAACCC	175
m_Col1a1	CCCTGGTCCCTCTGGAAATG	GGACCTTTGCCCCCTTCTTT	72
m_Mcp-1	CTTCTGGGCCTGCTGTTCA	CCAGCCTACTCATTGGGATCA	127
m_a-Sma	ACTACTGCCGAGCGTGAGAT	AAGGTAGACAGCGAAGCCAG	452
m_Shp	GGCACGATCCTCTTCAACCC	GGCACCAGACTCCATTCCAC	287
m_Fabp6	GAGACGTGATTGAAAGGGGA	TTACGCGCTCATAGGTCACA	294
m_Cyp7a1	CTGGGGGATTGCTGTGGTAG	CTGTGTCCAAATGCCTTCGC	315
m_Abcc2 (1)	TCCAGGACCAAGAGATTTGC	TCTGTGAGTGCAAGAGACAGG	107
m_Abcc4 (1)	TAATGGAAGCAGACAAGGCCCAGA	AGAGGCCAGTGCAGATACATGGTT	137
h_SHP (2)	CCCAAGATGCTGTGACCTTTGAG	TGGGGTCTGTCTGGCAGTTG	118
h_SLC51A (2)	TTCCTCTAAAACCAGGTCTCAAGT	GCACAGTCATTAGAAAAGTCTCCA	71
h_CYCLOA	ACGGCGAGCCCTTGG	TTTCTGCTGTCTTTGGGACCT	66 / 223

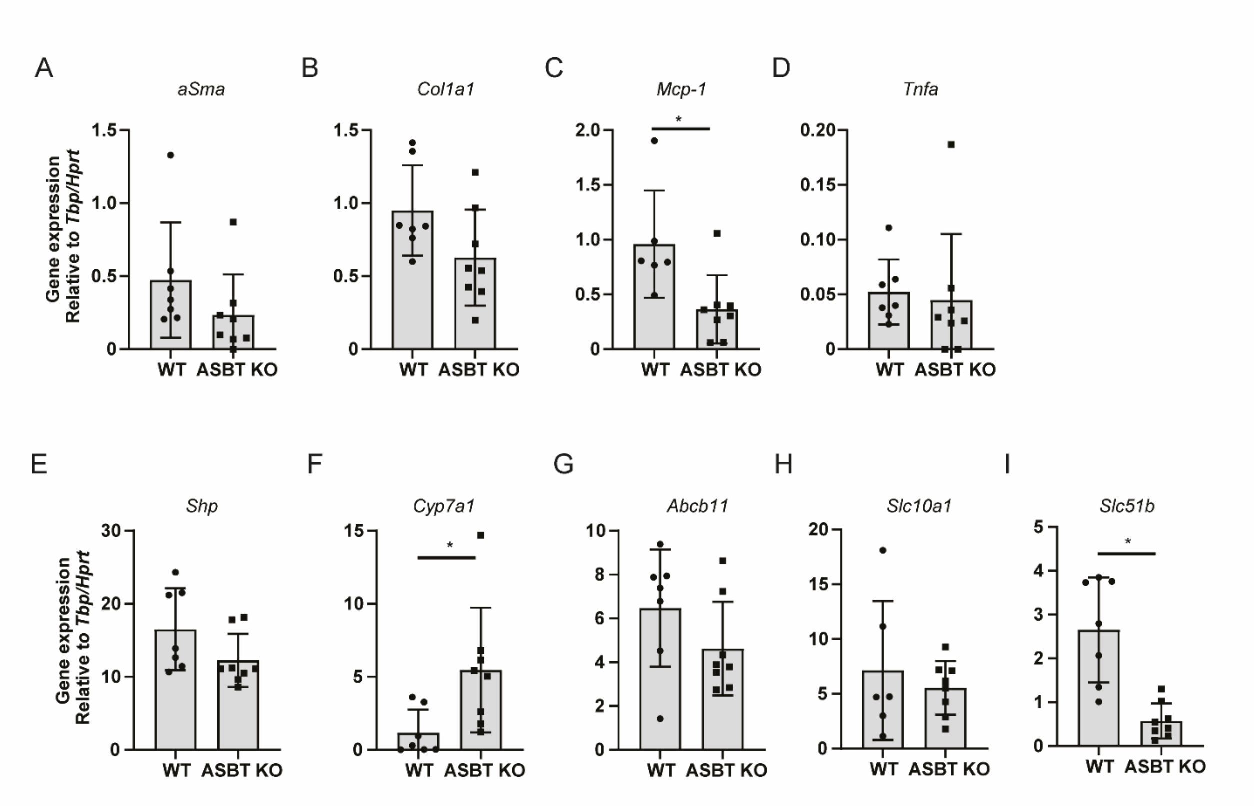

Fig. S1: Liver inflammatory genes are decreased in ASBT KO mice after BDL Relative mRNA expression of A) Liver *αSma*, B) Liver *Col1a1*, C) Liver *Mcp-1*, D) Liver *Tnfα*, E) Liver *Shp,* F) Liver *Cyp7a1*, G) Liver *Abcb11b,* H) Liver *Slc10a1*, I) Liver *Slc51b*. Individual values are shown ± standard deviation. *p<0.05

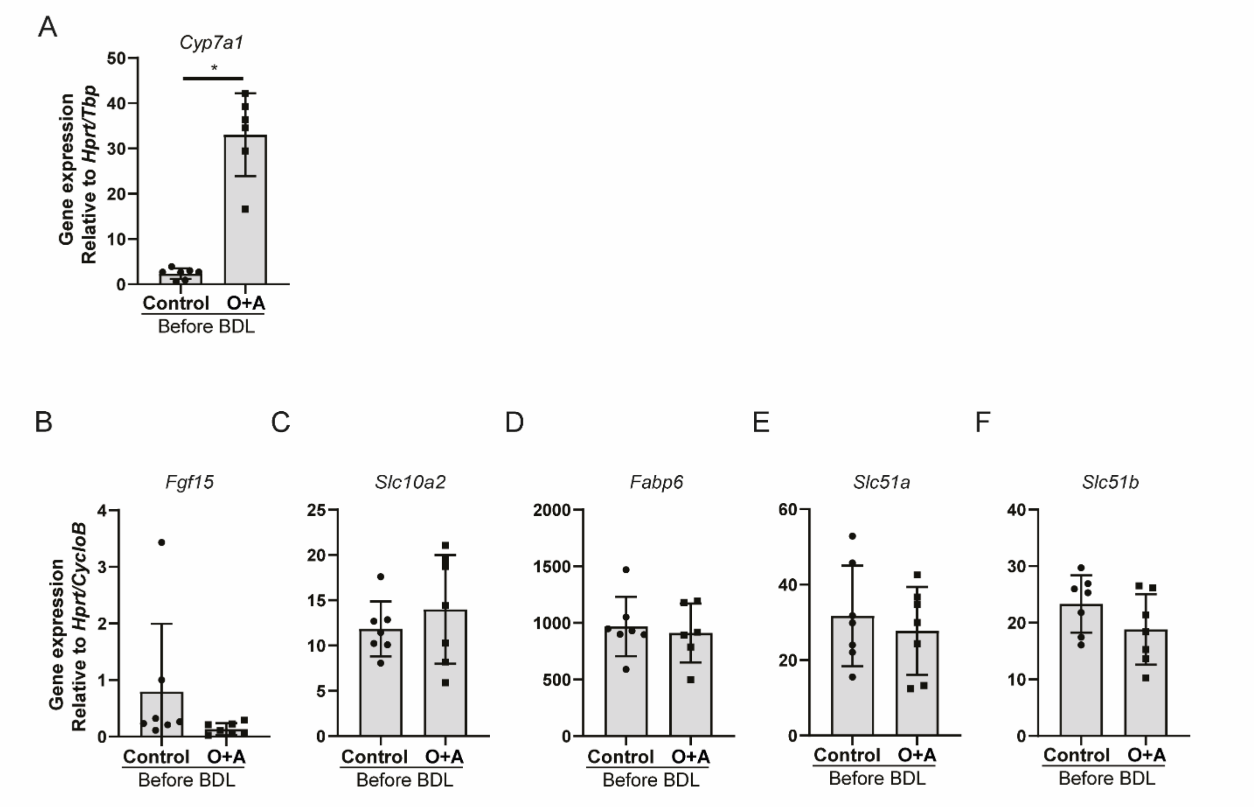

Fig. S2: Pre-treatment to reduce pool size before BDL does not affect ileum bile salt transport Relative mRNA expression of A) Liver *Cyp7a1*, B) Ileum *Fgf15*, C) Ileum *Slc10a2*, D) Ileum *Fabp6*, E) Ileum *Slc51a*, F) Ileum *Slc51b.* Individual values are shown ± standard deviation. $^{*}p<0.05$

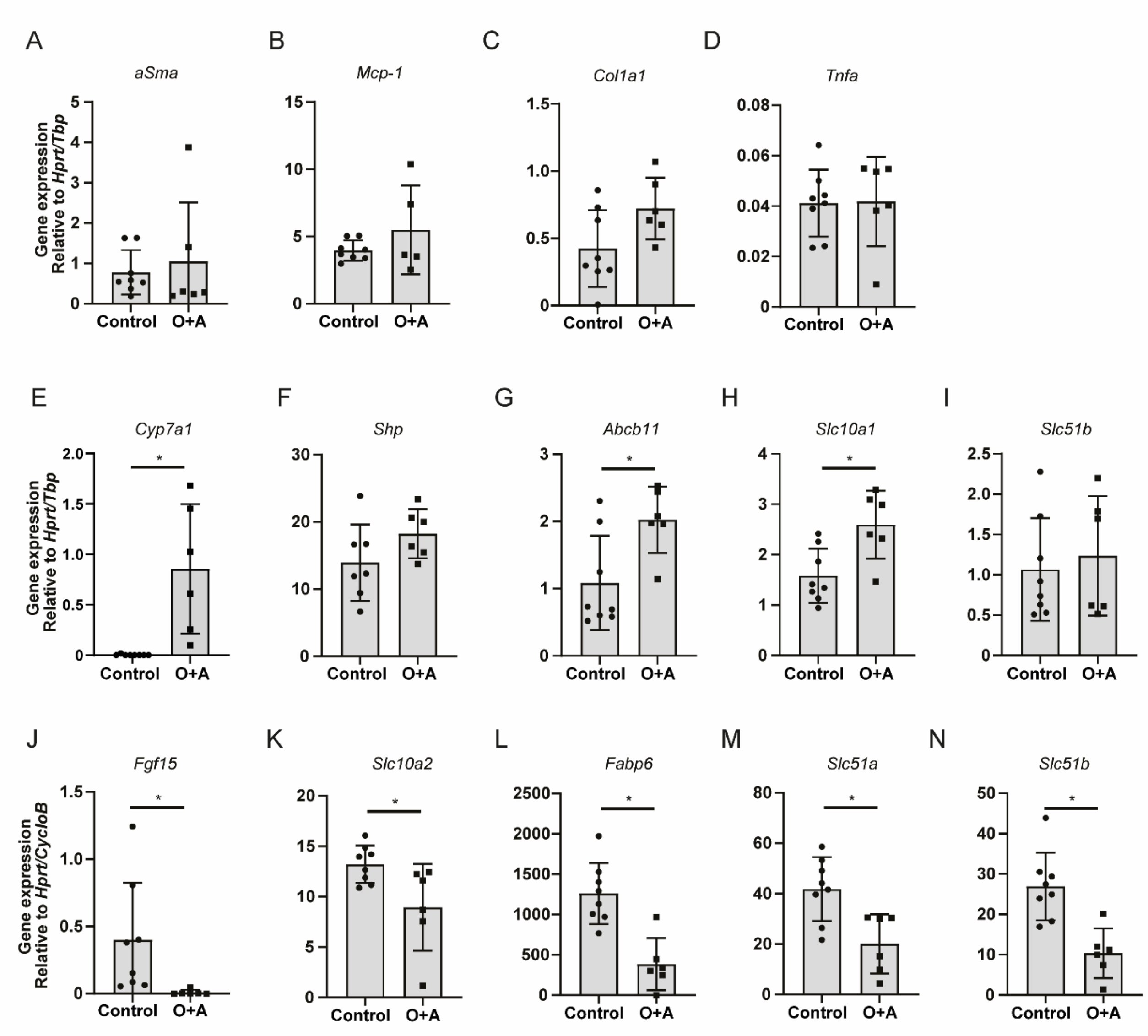

Fig. S3: Reduced pool size at cholestasis onset lowers ileal bile salt transport two-days after BDL Relative mRNA expression of A) Liver *αSma*, B) Liver *Mcp-1*, C) Liver *Col1a1,* D) Liver *Tnfα*, E) Liver *Cyp7a1*, F) Liver *Shp*, G) Liver *Abcb11b,* H) Liver *Slc10a1*, I) Liver *Slc51b* J) Ileum *Fgf15,* K) Ileum *Slc10a2*, L) Ileum *Fabp6*, M) Ileum *Slc51a* and N) Ileum *Slc51b*. Individual values are shown ± standard deviation. *p<0.05

Supplementary references

1. Flores, K., Manautou, J. E., & Renfro, J. L. (2017). Gender-specific expression of ATP-binding cassette (Abc) transporters and cytoprotective genes in mouse choroid plexus. Toxicology, 386, 84–92. https://doi.org/10.1016/j.tox.2017.05.019
2. Ijssennagger N, Janssen AWF, Milona A, Ramos Pittol JM, Hollman DAA, Mokry M, Betzel B, Berends FJ, Janssen IM, van Mil SWC, Kersten S. Gene expression profiling in human precision cut liver slices in response to the FXR agonist obeticholic acid. J Hepatol. 2016 May;64(5):1158-1166. doi: 10.1016/j.jhep.2016.01.016. Epub 2016 Jan 23. PMID: 26812075.
