# Supplementary material for: Systemic ASBT inactivation protects against liver damage in obstructive cholestasis in mice
Source: JHEP Rep. 2022 Aug 27;4(11):100573. doi: 10.1016/j.jhepr.2022.100573 (PMC9494276; doi:10.1016/j.jhepr.2022.100573)
Supplement: Multimedia component 2 [file mmc2.docx]

**JHEP Reports**

**CTAT methods**

Tables for a “Complete, Transparent, Accurate and Timely account” (CTAT) are now mandatory for all revised submissions. The aim is to enhance the reproducibility of methods.

- Only include the parts relevant to your study
- Refer to the CTAT in the main text as ‘Supplementary CTAT Table’
- Do not add subheadings
- Add as many rows as needed to include all information
- Only include one item per row

**If the CTAT form is not relevant to your study, please outline the reasons why:**

|  |
| --- |

- 1. **Antibodies**

| **Name** | **Citation** | **Supplier** | **Cat no.** | **Clone no.** |
| --- | --- | --- | --- | --- |
| **Anti-CK7** |  | **Abcam** | **ab181598** | **EPR17078** |

- 1. **Cell lines**

| **Name** | **Citation** | **Supplier** | **Cat no.** | **Passage no.** | **Authentication test method** |
| --- | --- | --- | --- | --- | --- |
| **HepG2** |  | **ATCC** |  |  |  |
| **U2OS** |  | **ATCC** |  |  |  |
| **U2OS_NucleoBAS** | **van der Velden, et al. (2013) Hepatology.** |  |  |  |  |
| **U2OS_HA_hNTCP** | **Bijsmans et al. (2012) Biochem J.** |  |  |  |  |
| **CCLP1** | **Shimizu et al. (1992) Int J Cancer** |  |  |  |  |

- 1. **Organisms**

| **Name** | **Citation** | **Supplier** | **Strain** | **Sex** | **Age** | **Overall n number** |
| --- | --- | --- | --- | --- | --- | --- |
| **ASBT KO mice and WT littermates** |  | **The Jackson Laboratory** | **129-Slc10a2tm1Pda/J** | **M/F** | **>8w** | **21 ASBT KO + 19 WT littermates** |
| **Wild type mice** |  | **Envigo** | **C57BL/6JOlaHsd** | **M** | **>8w** | **30** |

- 1. **Sequence based reagents**

| **Name** | **Sequence** | **Supplier** |
| --- | --- | --- |
|  |  |  |

- 1. **Biological samples**

| **Description** | **Source** | **Identifier** |
| --- | --- | --- |
|  |  |  |

- 1. **Deposited data**

| **Name of repository** | **Identifier** | **Link** |
| --- | --- | --- |
|  |  |  |

- 1. **Software**

| **Software name** | **Manufacturer** | **Version** |
| --- | --- | --- |
| **LinRegPCR** | **Ramakers et al. (2003) Neuroscience letters.** | **12.5** |
| **GraphPad** | **PRISM** | **9** |
| **Image J FIJI** | **National Institutes of Health** | **2.5.0** |
| **Bruker Compass** | **Bruker** | **6.0** |
| **LAS X** | **Leica** |  |

- 1. **Other (*e.g*. drugs, proteins, vectors etc.)**

| **OCA** | **Cayman Chemicals** | **11031** |
| --- | --- | --- |
| **G-OCA** | **Cayman Chemicals** | **28242** |
| **T-OCA** | **Cayman Chemicals** | **28243** |
| **ASBT inhibitor** | **GSK2299027B** | **Used for *in vivo* exp** |
| **pmKate2-N1** | **Evrogen** | **See manuscript** |
| **pSNAPm** | **New England Biolabs** | **See manuscript** |
| **Myrcludex B** | **PepScan** |  |
| **ASBT inhibitor** | **GSK264W94** | **Used for *in vitro* exp** |

- 1. **Please provide the details of the corresponding methods author for the manuscript:**

| **Stan van de Graaf, Ph.D.,**  **Tytgat Institute for Liver and Intestinal Research, Academic Medical Center, Meibergdreef 69-71, 1105 BK Amsterdam, The Netherlands.**  **E-mail: k.f.vandegraaf@amsterdamumc.nl**  **Tel: +31-020-5668832**  **Fax: +31-020-5669190** |
| --- |

**2.0 Please confirm for randomised controlled trials all versions of the clinical protocol are included in the submission. These will be published online as supplementary information.**

|  |
| --- |
